# Genetic Requirement for Pneumococcal Ear Infection

**DOI:** 10.1371/journal.pone.0002950

**Published:** 2008-08-13

**Authors:** Huaiqing Chen, Yueyun, Jun Yang, Christopher J. O'Brien, Scott L. Lee, Joseph E. Mazurkiewicz, Sauli Haataja, Jing-Hua Yan, George F. Gao, Jing-Ren Zhang

**Affiliations:** 1 Center for Immunology and Microbial Disease, Albany Medical College, Albany, New York, United States of America; 2 Division of Otolaryngology-Head & Neck Surgery, Albany Medical College, Albany, New York, United States of America; 3 Center for Neuropharmacology and Neuroscience, Albany Medical College, Albany, New York, United States of America; 4 Department of Medical Biochemistry and Molecular Biology, University of Turku, Turku, Finland; 5 Center for Clinical Molecular Biology, Xijing Hospital, Fourth Military Medical University, Xi'an, China; 6 Center for Molecular Immunology, Institute of Microbiology, Chinese Academy of Sciences, Beijing, China; The Research Institute for Children at Children's Hospital New Orleans, United States of America

## Abstract

**Background:**

Ear infection or otitis media (OM) accounts for most bacterial respiratory infections in children in both developed and developing nations. *Streptococcus pneumoniae*, nontypeable *Haemophilus influenzae*, and *Moraxella catarrhalis* are the major OM pathogens. However, little is known about the genetic basis of bacterial OM largely due to practical difficulties in conducting research in ear infection models and genetically manipulating clinical isolates. Here, we report the first genome-scale *in vivo* screen for bacterial genes required for ear infection in a chinchilla model by signature tagged mutagenesis (STM), a high throughput mutant screen technique.

**Methodology/Principal Findings:**

STM strains were constructed with a multi-drug resistant OM isolate ST556 (serotype 19F) and screened in a chinchilla OM model. Out of 5,280 mutants tested, 248 mutants were substantially underrepresented in the mutant pools recovered from the middle ear fluids of the infected chinchillas, indicating the impaired ability to survive and replicate in the middle ears due to genetic disruptions in the chromosome of strain ST556. Further DNA sequencing analysis mapped the mutations to 169 pneumococcal genes. Surprisingly, only 52 of these genes were required for pneumococcal nasopharyngeal colonization in a murine model. This infection site-specific gene requirement was verified by targeted mutagenesis in the selected genes.

**Conclusions/Significance:**

These findings suggest that there are a subset of pneumococcal genes required for ear infection and that these may be distinct from those required for nasal colonization. Our data thus provide comprehensive gene targets for mechanistic understanding of pneumococcal ear infection. Finally, this study has also developed a model for future genome-scale search for virulence determinants in other pathogens associated with ear infections.

## Introduction

Acute otitis media (OM) accounts for most bacterial respiratory infections in children in both developed and developing nations [1]. Recent surveys suggest that OM is the most common cause for visits to the emergency room and the second ranking reason for visits to a physician's office in the United States (more than 20 million per year) [2]. By three years of age, 80 percent of all children in the United States have had at least one episode of OM, and 50 percent have had at least three episodes; the estimated annual cost associated with OM is over 5 billion dollars in the United States alone [1]. OM is characterized by excessive mucus secretion and inflammation with an infiltration of the subperiosteal space by leukocytes, macrophages and mast cells [1]. In contrast to the significant impact of the OM on public health, the pathogenic mechanisms of OM are largely unclear. Some bacterial factors have been identified to contribute to the OM pathogenesis, but no study has been conducted to identify virulence determinants at a genome scale. This situation is partly caused by limited availability of suitable and convenient animal models [3].

Infections caused by *Streptococcus pneumoniae*, nontypeable *Haemophilus influenzae*, and *Moraxella catarrhalis* account for the majority of OM [4]. Among the bacterial pathogens, *S. pneumoniae* is the most common cause of OM, accounting for approximately 40% of the episodes [4]. *S. pneumoniae* is naturally able to colonize in the human nasopharynx, subsequently causing infection in the middle ear and other remote tissues [5]. Based on the structural and antigenic variation of the capsular polysaccharide, *S. pneumoniae* isolates have been classified into 91 serotypes [6], [7]. OM is predominately associated with strains from serotypes 3, 6A, 6B, 9V, 14, 19A, 19F, and 23F [8]. *S. pneumoniae* is also an important pathogen causing pneumonia, bacteremia, and meningitis. Treatment of pneumococcal infections depends on the administration of antibiotics, but the systematic use of antibiotics has led to the rapid emergence of multi-drug-resistant pneumococcal isolates [9]. The current capsular polysaccharide-based vaccines have limited efficacy against pneumococcal OM and other infections [1].

Previous studies have identified many virulence-associated factors for pneumococcal nasal colonization, pneumonia, and bacteremia [5]. In particular, three studies have used signature-tagged mutagenesis (STM) to identify a large list of pneumococcal genes that are required for bacterial survival in murine models of pneumonia [10]–[12] and bacteremia [11]. In sharp contrast, very few of the virulence factors identified in other pneumococcal infection models have been studied in OM models [13], [14]. The *nanA* gene encoding the major neuraminidase of *S. pneumoniae* is required for ear infection in the chinchilla model [13]. STM was originally developed to identify virulence factors in *Salmonella typhimurium* by negative selection [15]. This technique has been successfully used to identify virulence-associated genes in many other pathogens [16]. In this study, we performed an STM screen with a low-passage clinical isolate in a modified OM chinchilla model. This work led to the identification of 169 bacterial genes that are potentially required for ear infection. We further determined that the majority of the genes are not required for pneumococcal nasal colonization, suggestive of tissue-specific genetic requirement for ear infection.

## Results

### Establishment of middle ear infection screen model in chinchillas

**Figure 1:**
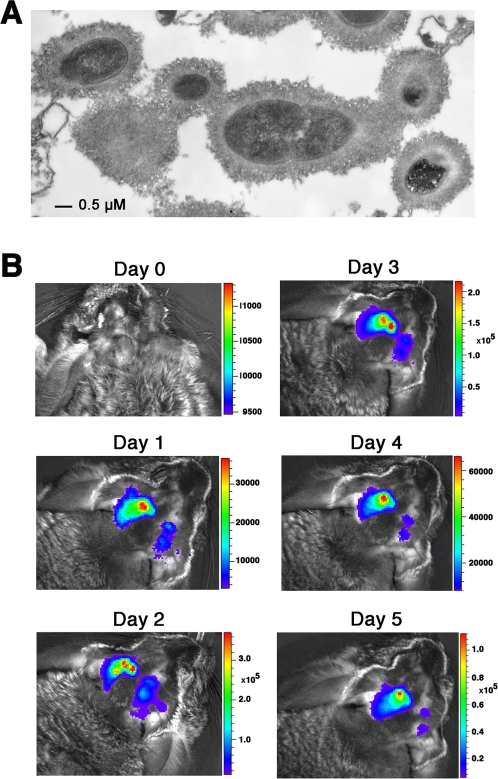
Middle ear infection by pneumococcal strain ST556. (A) Detection of ST556 encapsulation by transmission electron microscopy. (B) Detection of ST556 infection in the middle ears. Chinchillas were infected with bioluminescent pneumococci via tympanotomy and monitored daily for bacterial burden by bioluminescence. The scales on the right indicate the levels of photon counts.

To identify pneumococcal factors that are specifically associated with OM, we chose to study a low-passage multi-drug resistant serotype 19F isolate ST556 [8]. 19F is one of the few serotypes that are commonly associated with OM [8], [17]. We first sought to verify capsule production of ST556 because the capsule is an essential virulence factor for pneumococcal nasal colonization, pneumonia, and bacteremia [5]. Electron microscopy analysis showed that ST556 is indeed encapsulated (Fig. 1A). We also found that ST556 is transformable by natural transformation, which was essential for preparing tagged mutants for *in vivo* screen. Another key requirement for successful STM screen is the ability to recover sufficient numbers of inoculated bacteria from live animals post infection to allow the identification of each representative mutant that survives within a mutant pool [16]. To this end, it is critical to select an appropriate infection model and parameters. Various animal models including chinchilla and mouse have been used to study middle ear infection of *S. pneumoniae* [18]. However, no ear infection models had been used for a high throughput STM screen. Given its large space and accessibility of the middle ear, chinchillas were assessed for their suitability for our STM screen.

To assess bacterial burden profiles at various stages of pneumococcal infection in the middle ears by a noninvasive bioluminescent imaging approach [19], we first engineered a bioluminescent ST556 derivative that carried the *luxCDABE* gene cluster of *Photorhabdus luminescens.* The middle ears of chinchillas were then infected with various doses of the bioluminescent pneumococci via tympanotomy. As represented in Fig. 1B, pneumococci in the middle ear of infected chinchillas became detectable 24 hr post infection and remained detectable for at least 5 days. Consistent with the nontypeable *H. influenzae* study [19], the detection limit of this technique was approximately 10^5^ colony-forming unit (CFU) for *S. pneumoniae* in the middle ears as determined by CFU enumeration (data not shown). As judged by bioluminescence level and CFU count, bacterial burden reached the highest level (>10^7^ CFU/ear) around 3 days post infection (Fig. 1B). This observation was consistent with the progression of bacterial burdens in human ear infections in the absence of antibiotics [20]. All chinchillas were able to survive the infection with up to 2×10^4^ CFU of ST556 (data not shown). This resistance level allowed relatively large infection doses of the mutant pools thereby maximizing the representation of each mutant in the inoculums. We also found that the uninoculated ears of the animals infected through the other ear mostly remained sterile or contained low levels of the inoculated pneumococci. This observation allowed us to use both ears of a single animal to screen the STM strains and thus minimized the number of animals associated with our STM screen. To further test the feasibility of this model, we separately infected the left and right ears of two chinchillas with the same pool of 55 STM strains (10^4^ CFU pneumococci/ear; 182 CFU/mutant/ear). We consistently recovered high levels of the bacterium (>10^5^ CFU/ear) from the middle ear washes of all infected animals 3 days post infection with minimal mortality. Finally, the mutants that were lost or underrepresented in one ear were virtually always lost or underrepresented in the other ear of the same chinchilla. Therefore, the “bottleneck” issue encountered by Hava *et al*. [10] in the lung infection model did not appear to play an appreciable role in this ear infection model.

### Construction of STM strains in *S. pneumoniae* ST556

**Figure 2:**
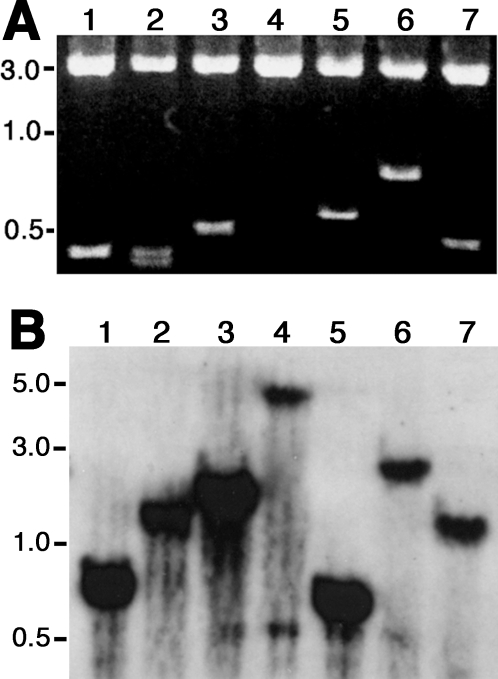
Construction of STM strains in ST556. (A) Sequence diversity of the STM constructs. The pneumococcal DNA inserts from 7 *E. coli* transformants (1–7) were released from the pID701t plasmid by restriction digestion with *Xho*I and *Xba*I, which flanked the original *Sma*I cloning site. DNA standards are indicated in kilobase (kb). (B) Random plasmid insertions in ST556 chromosome. Genomic DNA preparations from seven ST556 STM strains (1–7) were digested by *Hind*III, separated by agarose gel electrophoresis, and hybridized with plasmid-specific probe.

Tagged mutants of *S. pneumoniae* ST556 were generated by insertion duplication with derivatives of the pID701t suicide plasmid that were used in a previous STM study with a type-3 *S. pneumoniae* strain [11]. As shown in Fig. 2A, randomly selected clones of the *E. coli* transformants covered a wide range of pneumococcal chromosomal loci as reflected by various sizes of DNA inserts after restriction digestion. The recombinant plasmids were transformed into *S. pneumoniae* ST556 to generate disruptive mutations. The Southern blot analysis of selected pneumococcal mutants indicated that the tagged plasmids were inserted into the chromosome of strain ST556 in a highly random manner (Fig. 2B). A total of 5,280 STM strains were arranged in 96 mutant sets (∼55 mutants/set) based on the identity of the sequence tags to prepare the input pools.

### Negative selection and sequence identification of attenuated mutants in the OM model

**Figure 3:**
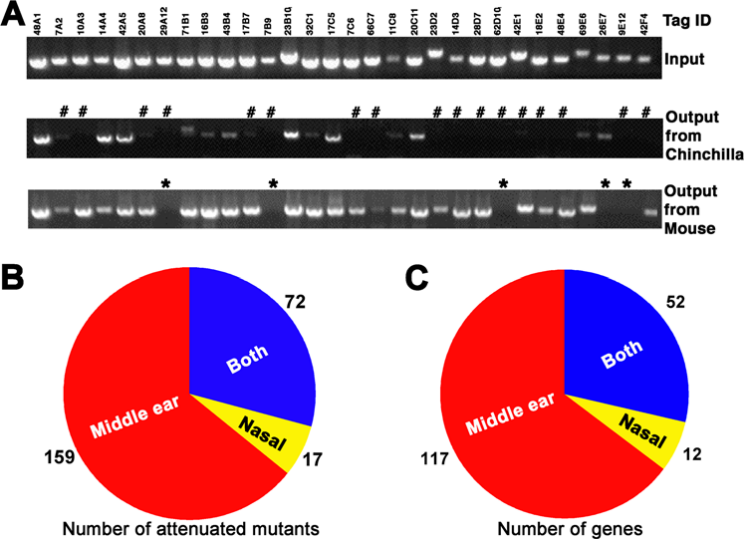
Negative selection of the STM strains. (A) Detection of the attenuated STM strains. The same input pool #3 was used to infect the middle ears of chinchillas or nasopharynx of mice in the 2^nd^ round screen. The animals were sacrificed 3 days post infection to recover the bacteria (output pool) from the middle ears (chinchillas) or nasopharynx (mouse). The input and output pools were compared by PCR amplification and agarose gel electrophoresis. Pound signs (#) and asterisks (*) indicate the mutants that were missing in the output pools from the middle ear and nasopharynx, respectively. (B) Schematic representation of the STM strains identified in the 2^nd^ round screen. The red and yellow colors indicate the total numbers of the mutants that were attenuated only in the middle ear infection or nasal colonization, respectively. The mutants attenuated in both models are represented by blue color. (C) Schematic representation of the genes identified in the 2^nd^ round screen as in (B).

The input pools were inoculated into the middle ears of chinchillas via tympanotomy and selected under the conditions defined in the preliminary trials. The remaining bacteria (output pools) were recovered 3 days later by ear lavaging to detect the presence or absence of individual mutants by polymerase chain reaction (PCR) using tag-specific primers (Fig. 3A). This procedure identified 678 strains that were completely missing or substantially underrepresented in the PCR profiles. To exclude the mutants with marginal attenuation, the resulting mutants were then arranged into 20 sets of 29–32 mutants, and subjected to a 2^nd^ round of screen in chinchillas. A total of 248 mutants were found to be completely missing in the 2^nd^ round screen. These strains are referred to as attenuated mutants hereafter. Plasmid insertion sites of the mutants were mapped to 169 genes by DNA sequencing (Table 1; Table S1). This represents ∼9% of the predicted *S. pneumoniae* open reading frames (ORF) based on the fully sequenced genome of TIGR4 [21]. Since the ST556 genome has not yet been determined, the genes identified in this work are organized according to the gene annotations of strain TIGR4. This arrangement has also allowed comparative analysis of our data with those from the TIGR4 STM study [10]. Allelic homologs were found for the majority of the genes (132, 78%) in the TIGR4 genome, whereas 32 genes are not shared by TIGR4 (Table S1). We refer to these as ST556-specific genes in this context.

**Table 1:** Functional classification of pneumococcal genes identified in this study

Category	No. of Genes	Functional description
Transport	34	Carbohydrates, amino acids, ion, nucleotides, and others
Cellular processes	22	House-keeping enzymes/proteins
Nucleic acid processing	13	Enzymes/proteins involved in DNA replication/repairing and DNA/RNA cleavage
Transcription	12	Proteins associated with basic transcription and transcriptional regulation
Translation	9	Enzymes/proteins involved in biosynthesis of amino acid-charged tRNAs and protein synthesis
Biosynthesis	8	Amino acids, nucleic acids, and fatty acids
Surface	7	Proteins associated with cytoplasmic membrane and cell wall
Cell division	4	Proteins required for cell separation and shape
Energy metabolism	3	Enzymes associated with ATP synthesis and related functions
Drug resistance	1	Tetracycline resistance protein TetM
Unknown	66	Lack of defined functions with or without TIGR4 homologs
Total	169	

Consistent with other STM studies [10], [11], a large portion of the identified genes (34; 20.1%) represents transport-associated functions, many of which encode ABC transporters for carbohydrates and amino acids (Table 1). As anticipated, some of the genes (22, 13%) encode various enzymes that are associated with cellular processing functions. In agreement with the observation by Hava *et al.* [10], 12 genes (7%) are involved in transcription. All of the mutants in this category carried mutations in the genes encoding transcription factors except strain 94E07, which harbored an insertion in the 3′ end of *rpoD* (encoding sigma-70 subunit of RNA polymerase). To our surprise, some of the attenuated mutants represent the genes encoding 6 aminoacyl-tRNA synthetases, which include leucyl-tRNA synthetase that was previously identified in the TIGR4 STM study in a lung infection model [10]. This study also identified several surface-associated proteins including pneumococcal surface protein A (PspA) and choline binding protein A (CbpA). PspA and CbpA are well-described virulence factors for nasopharyngeal colonization and lung infection of *S. pneumoniae* [22]–[24]. Both PspA and CbpA were also found in the lung infection STM study with strain TIGR4 [10], but this is the first study showing that these factors are also required for ear infection. Finally, the largest class of the genes (66, 39%) has no defined functions. Virtually all of the ST556 unique genes (35 out of 37) fall into this category. These include OM-associated pneumococcal sequences P23 (TIGR4 SP405) and H10 (absent in TIGR4) recently identified by subtractive hybridization [25]. As reported in other pneumococcal STM studies [10], [11], we did not identify any mutants that represent the capsule biosynthetic gene cluster.

### Identification of pneumococcal genes only required for ear infection

Previous STM studies have indicated that certain pneumococcal genes are only required for infection in a host tissue/organ-specific fashion [10]–[12]. We thus tested whether all of the genes identified from the OM model are required for pneumococcal nasal colonization. We initially tested suitability of a chinchilla model for pneumococcal nasopharyngeal colonization as previously described by Chen *et al.* [26]. However, the pneumococci recovered from the nasal washing of chinchillas infected by ST556 derivatives were frequently experimentally contaminated by indigenous microorganisms since these animals were typically maintained in commercial ranches instead of the SPF conditions where laboratory mice are typically raised. In addition, there was a greater level of animal-to-animal inconsistency in terms of total levels of the recovered pneumococci as compared with the mouse model. These limitations prompted us to use a mouse model for pneumococcal colonization in this analysis [27]. The mutants identified in the 1^st^ round screen with the OM model were “re-screened” in a murine model of nasal colonization with the same mutant pools used for the 2^nd^ screen in the OM model (Fig. 3A). To our surprise, this screen identified only 89 attenuated mutants with disruptions in a total of 64 genes, whereas the majority of the attenuated mutants in the OM model were readily detectable in the output pools from the nasal lavages (Fig. 3B; Table S1). This screen also identified 12 genes that are only required for nasal colonization but not for ear infection (Table S2). This skewed result was due to the fact that the mutants had been prescreened in the OM model. Together, these data show that we have identified a large number of OM-specific pneumococcal genes.

### Confirmation of attenuation phenotype of selected mutants

**Figure 4:**
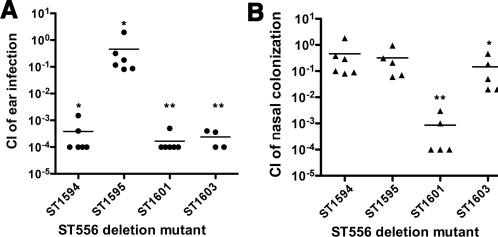
Quantification of the attenuation with ST556 deletion mutants. (A) Middle ear co-infection. Both the left and right ears of chinchillas (n = 3) were infected by tympanotomy with a 1∶1 mixture of the wild type and isogenic deletion mutants ST1594 (SP0308), ST1595 (*tetM*), ST1601 (SP1732, *stkP*) or ST1603 (SP1448). Each symbol represents the CI value from a single ear of infected chinchillas. CI is defined as the output CFU ratio (mutant/wild type) divided by the input CFU ratio (mutant/wild type). Bar indicates the geometric mean CI value for each group of chinchillas. Statistical significance was determined by Student's *t* test. *, *P*<0.05, **, *P*<0.01. (B) Nasal co-infection. BALB/c mice (n = 5) were intranasally infected as in (A). The data were analyzed and presented as in (A).

To verify the STM screen data, we generated deletion mutations in SP0308 (transport), SP1448 (unknown), and *tetM* (drug resistance) of strain ST556 by allelic replacement. The STM strains representing these genes showed impaired fitness in the middle ear but not during nasal colonization (Table S1). The resulting mutants were used to perform co-infection experiments in both OM and nasal colonization models. An isogenic ΔSP1732 mutant ST1601 was used as positive control for nasal colonization since our preliminary study showed that this mutant was deficient in nasal colonization (data not shown). SP1732, encoding an eukaryotic-type membrane-associated serine/threonine protein kinase [28], is required for pneumococcal virulence [29], [30]. All mutants displayed a similar growth rate as the wild type ST556 in THY broth (data not shown). Bacteria were enumerated from the ear fluid and nasal washing samples 3 days post infection. In line with the STM screen results, the ΔSP0308 (ST1594) and ΔSP1448 (ST1603) mutants were attenuated by >1,000 fold in the middle ear (Fig. 4A) but relatively normal in the nasal colonization (Fig. 4B). Contrary to the STM results, the Δ*tetM* mutant ST1595 showed only moderate defect in both infection models. This discrepancy may be due to a polar effect in the STM strains. Consistent with previous studies [29], [30], the ΔSP1732 mutant showed significant deficiency in both models. It should be mentioned that we did not identify a SP1732 mutant in our STM screen, thus indicating that our screen did not saturate the ST556 genome. The co-infection experiments thus validated our STM results that certain *S. pneumoniae* genes are only required for ear infection.

As an effort to verify the essentiality of the potentially essential genes identified in our screen, we attempted to delete each of the six aminoacyl-tRNA synthetase genes for arginine, cysteine, histidine, lysine, leucine, and threonine in strain ST556 by allelic exchange with the Janus cassette. The arginyl-tRNA synthetase gene (*argS*) appears to be essential since we failed to obtain any viable *argS* deletion mutants in multiple trials. However, we have successfully obtained deletion mutants with the other five genes. All of these mutants were able to grow in THY broth, but the growth rates were noticeably slower than that of the wild type strain (data not shown). To further characterize these mutants, primers representing the flanking regions of the target genes were used to amplify the Janus Cassette by PCR. Restriction digestion and DNA sequencing analysis of the PCR products showed that the lysyl-tRNA synthetase gene (*lysS*) was completely replaced by the Janus cassette (data not shown). Interestingly, a second PCR amplicon was always present in each of the *cysS*, *hisS*, *leuS*, and *thrS* mutants (represented in the supplemental Figure S1). This amplicon in each of the mutants was always similar in size to that of the corresponding wild type gene, suggesting the presence of both the truncated and wild type aminoacyl-tRNA synthetase genes in each of these mutants. To rule out the possibility of mixed clonal populations in the mutant stocks, we isolated single colonies for each of the *cysS*, *hisS*, *leuS*, and *thrS* mutants. The DNA sequencing analysis revealed the presence of the Janus Cassette in each of these five aminoacyl-tRNA synthetase genes. However, the repeated experiments consistently showed the “wild type-like” PCR products in all of the clonal populations. The precise nature of this observation remains to be defined. Together, while *argS* appears to be essential, deletion mutants for *lysS, cysS*, *hisS*, *leuS*, and *thrS* could be obtained.

## Discussion

This study represents the first extensive search of bacterial factors that are required for ear infection. Our STM screen identified a total of 169 genes that are potentially required for ear infection from a pneumococcal OM isolate. These include a set of genes that were previously found to be important for pneumococcal infections in other tissues. In particular, 23 genes described here were discovered in at least one of the three previous STM studies using murine models of pneumonia [10]–[12] and bacteremia [11] (Table S1). Some of these genes represent well-characterized virulence determinants such as CbpA, PspA, and RlrA. CbpA and PspA are the surface-exposed proteins that have been implicated as bacterial complement-resistance factors [24], [31]–[33] or adhesin [22], [34]–[36]. RlrA is a transcriptional activator for the *rlrA* pathogenicity islet [37], which encodes the recently described pneumococcal pilus [38], [39]. Although the *rlrA* pathogenicity islet is only present in some pneumococcal strains/clones [40], our preliminary sequencing analysis identified all of the seven genes of this locus in the ST556 genome including *rlrA, srtD* and *rrgA* (unpublished data). *srtD* encodes a sortase that incorporates the RrgA protein into the pilus [38], [39]. Interestingly, an *srtD* mutant (strain 29E04) was also attenuated in our screen. Nelson et al. recently showed that the pilus promotes pneumococcal adhesion [41]. These lines of evidence strongly suggest that effective adhesion and tolerance to complement-mediated host defense are common requirements for bacterial infections in the middle ear and lung.

Our data suggest that genetic requirement for pneumococcal ear infection greatly differs from that of nasal colonization in animal models. Only 31% of the genes identified in the ear infection screen were “rediscovered” in the nasal colonization model. The majority of the identified genes should reflect the different selection pressure between the middle ear and nasopharynx. Tissue/disease-specific pneumococcal genes were also found in other STM studies [10]–[12]. Lau et al. performed parallel screens of the same type-3 mutant pools in murine models of pneumonia and bacteremia, and found that only a third of the attenuated strains were shared by both infection models [11]. The OM-specific genes may be required for pneumococcal fitness in terms of bacterial replication and survival in the middle ear. Some of these genes may be required for acquiring certain nutrients that are uniquely present in the middle ear. In agreement with this notion, the majority of the transport-related genes identified in this study have not been reported in the previous STM screens with the lung and bacteremia infection models. Unlike the relative quiescent host response to colonizing pneumococci at the nasopharynx [42], pneumococcal replication in the middle ear typically induces strong inflammation (e.g. mucosal secretion and recruitment of leukocytes) [43]. Even under these adverse conditions, *S. pneumoniae* is able to persist at high levels in the middle ears of humans and animal models [18], [20]. In this context, some of the OM-specific genes described here, such as those encoding transcriptional factors, are likely to participate in bacterial adaptation in the middle ear. Lastly, some of the differences might be caused by the genetic differences between the chinchilla and mouse infection models. It would be ideal to use a singe animal model but the inherent limitations for the chinchilla nasal colonization model and mouse ear infection model have prevented us from conducting these screens in the same animal model.

The majority of the genes described here have not been reported in the previous STM studies using murine models of pneumonia [10]–[12] and bacteremia [11]. Due to the lack of complete sequence information for the type-3 [11] and type-19 [12] studies, we can only perform comparative analysis with the type-4 STM screen in the murine pneumonia model [10]. Although both studies used similar numbers of STM strains, there is only a 25-gene overlap between the two screens (Table S1). As discussed above, the outcomes of these studies have highlighted the unique challenges that *S. pneumoniae* must face in the lungs and middle ears. Some of the variations could be caused by genetic variations between model animals and genome plasticity between strains ST556 and TIGR4. This is exemplified by the identification of many ST556 genes that are absent from the TIGR4 genome. A recent multi-genome comparison reveals that >50% of pneumococcal genes are not present in all strains [44]. Additional differences could be related to technical differences in two screens including the methods of mutagenesis (insertion duplication vs. transposon insertion) and mutant detection (PCR vs. Southern hybridization). Finally, the precise outcome of an STM screen also depends on the cutoff stringency since neither PCR- nor Southern hybridization-based detection method is fully quantitative.

A list of presumptive essential genes were identified in our screen such as those encoding the sigma 70 subunit of RNA polymerase (*rpoD*) and aminoacyl-tRNA synthetases. The attenuation phenotype of the *rpoD* mutant might be due to partial loss of sigma-70 function since the insertion only truncated 67 nucleotides from the 3′ end of the coding region. Alternatively, this could be due to a polar effect on the downstream genes since *rpoD* is in the middle of an apparent operon. Most of aminoacyl-tRNA synthetases are essential for bacterial survival and therefore considered to be promising drug targets [45]. However, some aminoacyl-tRNA synthetase functions can be compensated by biochemical modification of tRNA charged with structurally related amino acids (e.g., glutamate and glutamine) [46]. Some bacteria can possess multiple enzymes to synthesize the same aminoacyl-tRNAs as exemplified by the presence of two methionyl-tRNA synthetases [47]. None of the aminoacyl-tRNA synthetases appear to have a functional homolog in the TIGR4 genome. We are sequencing the ST556 genome to discern these possibilities. Finally, it is possible that the plasmid insertions in these aminoacyl-tRNA synthetase genes did not completely abolish the activities of these enzymes. This could explain the result with the STM strains of the *argS* gene since the arginyl-tRNA synthetase appears to be essential based on our failure to obtain viable *argS* deletion mutants. It is intriguing that the deletion mutants of the *cysS*, *hisS*, *thrS*, and *leuS* genes appeared to carry both the truncated and wild type alleles. We are in the process of characterizing these mutants. It should be noted that pneumococcal leucyl-tRNA synthetase was also identified by Hava *et al.* [10].

Some of the well-documented virulence factors for pneumococcal colonization and invasive infections were not represented in our screen. This can be explained by multiple possibilities. Some of the misses might be due to the incomplete coverage of STM genomes by our STM library. It is also possible that some of the virulence factors identified in other infection models are not required for ear infection as suggested by our data. Lastly, the lack of other genes could be caused by the defective growth of the relevant mutants. As reported with strain TIGR4 [10], we found that the capsule biosynthesis (*cps*) locus of ST556 is refractory to insertional mutations (unpublished observation). This may explain why we did not identify a single mutant in the capsule gene cluster. Some of the ST556 STM mutants were excluded from this study simply due to their extremely poor growth. Along this line, other complementary approaches are highly desirable to study those conditionally essential genes.

The OM model used in this study can be readily adapted to identify bacterial genes required for ear infection caused by nontypeable *H. influenzae* and *M. catarrhalis.* A limitation of this model is that direct inoculation of bacteria into the middle ears across the tympanic membrane bypasses the early dissemination stage from the nasopharynx. Therefore, the genes that are involved in the bacterial dissemination process can be missed. However, this approach will ensure the same start point for all mutants such that factors associated with bacterial fitness in the middle ears are equally represented in the screen procedure. Together, this study has provided valuable information for future investigations in genetic basis of ear infections caused by *S. pneumoniae* and other bacterial pathogens.

## Materials and Methods

### Bacterial strains and cultivation

ST556 is a serotype-19F isolate from a patient with otitis media [8]. ST556 was sensitive to chloramphenicol and kanamycin but resistant to penicillin and tetracycline. Pneumococci were routinely grown in THY broth or on tryptic soy agar (TSA) plates containing 3% (v/v) sheep blood in the presence or absence of indicated antibiotics as described [48]. *E. coli* cultures were grown in Luria-Bertani (LB) broth or on LB agar plates. All ingredients for bacterial culture media and other chemicals used in this work were obtained from Sigma (St. Louis, MO) unless otherwise stated.

### Characterization of strain ST556

Pneumococcal growth in the middle ear was monitored by non-invasive imaging of bioluminescent bacteria in live chinchillas (*Chinchilla lanigera*) with a Xenogen IVIS system (Alameda, California) as described [19]. One-year-old female chinchillas (Ryerson, Plymouth, OH) were infected with 10^4^ CFU of bioluminescent strain ST1493, a ST556 derivative carrying the *luxCDABE* genes of *Photorhabdus luminescens* by tympanotomy [49]. The *luxCDABE* was PCR amplified with primers Pr1393 and Pr1394 from genomic DNA of Xenogen Bioware strain Xen-11. The primers used in this work are described in the Supplemental Information (Table S3). Pneumococcal genomic DNA was prepared by phenol-chloroform extraction as described [48]. Bioluminescent pneumococci were generated by transforming ST556 with the gel-purified PCR product as described [48]. The kanamycin-resistant transformants (400 µg/ml) were further tested for bioluminescence with the IVIS system instrument according to the manufacturer's instructions. The ST556 capsule was visualized by transmission electron microscopy as described [50].

### Construction and *in vivo* selection of pneumococcal STM strains

Derivatives of the suicide pID701t plasmid were used to construct disruptive mutants in strain ST556 by insertion duplication essentially as described [11]. Each of the 55 plasmids used in this study contained a 52- base pairs (bp) unique tag sequence. ST556 genomic DNA was sheared by ultrasonicaton; DNA fragments of 300–700 bp were isolated by standard sucrose density gradient ultra-centrifugation. The DNA fragments were treated with T4 DNA polymerase and ligated into the *Sma*I site of pID701t. All molecular biology reagents were purchased from New England Biolabs (Beverly, MA). The resultant plasmids were transformed into *E. coli* DH5α and selected for chloramphenicol resistance (25 µg/ml). *E. coli* transformants harboring the same tags were pooled to prepare plasmids for transformation of ST556 by natural transformation [48]. Pneumococcal transformants were selected on TSA plates containing chloramphenicol (6 µg/ml) and assembled in 96 sets so that each of ∼55 mutants in a single set was uniquely tagged. The randomness of plasmid insertions in ST556 chromosome was assessed by Southern hybridization with genome DNA preparations of randomly selected STM strains as described [48].

Southern hybridization with genome DNA preparations of randomly selected STM strains was carried out as described [48]. Restriction enzyme-digested genomic DNA fragments were separated by agarose gel electrophoresis and visualized by staining agarose gels with ethidium bromide (2 µg/ml). A plasmid-specific probe representing the pID701t plasmid was amplified with primers Pr213 and Pr214 and the PCR DIG Probe Synthesis Kit as described by the supplier (Roche, Indianapolis, IN). Sizes of DNA fragments were estimated based on DIG-labeled DNA molecular weight standards (Roche).

The ST556 mutants were screened in a chinchilla model of OM [18]. All animal infection procedures were in compliance with the guidelines of the Institutional Animal Care and Use Committee (IACUC). The input pools of the 1^st^ round screen were prepared essentially as described [51]. The tagged ST556 mutants were assembled in 96 sets of 55 mutants based on the sequence identity of the tags. Approximately 10^4^ CFU of each input pool were used to infect both ears of two chinchillas by injecting 100 µl of the input pool suspension across the tympanic membrane using 27-gauge insulin syringes. The chinchillas were anesthetized with intramuscular injection of ketamine HCl (12 mg/kg) and xylazine (1.6 mg/kg) in PBS. Portions of the inoculums were diluted in PBS and spread on TSA blood plates containing chloramphenicol (6 µg/ml), ampicillin (1 µg/ml) and gentamycin (2 µg/ml) to verify the infection dose and to generate input pool DNA templates for subsequent PCR detection of tagged mutants. The middle ears were monitored daily for signs of bacterial infection by otoscopy. The animals were sacrificed 3 days post infection to recover the pneumococci by washing each of the middle ears with 300 µl PBS. The lavages were partially plated on TSA blood plates containing chloramphenicol to establish the output pools. The bacteria in the output pools were grown in THY to prepare genomic DNA for subsequent PCR detection of tagged mutants. The mutants identified in the 1^st^ round screen were arranged into pools of ∼30 mutants for a 2^nd^ round screen in both the chinchilla and mouse models as described above.

The screen experiments in the murine nasal colonization model were carried out essentially as described [27]. Groups of three female BALB/c mice from Taconic Farm (6–8 weeks old; Germantown, NY) were each infected with a single input pool by intranasal inoculation of 20 µl bacterial suspension (∼10^5^ CFU). The mice were anesthetized with ketamine HCl (Fort Dodge Animal Health, Fort Dodge, Iowa) and xylazine (Phoenix Scientific, St. Joseph, Mo.) in PBS immediately prior to infection. Three days later, mice were sacrificed to collect nasal lavages from each of infected mice by retro-tracheal washing with 500 µl PBS, and plated on antibiotic-containing THY agar dishes to prepare output pools as described above. We typically recovered >10^5^ CFU of pneumococci from each ear of infected chinchillas and >10^4^ CFU from each infected mouse 3 days post inoculation. We repeated all infection experiments that either yielded low numbers of bacterial colonies in the output pools (<10^4^ CFU) or highly contaminated output colonies as assessed by colony size and morphology.

### PCR detection and sequence identification of the STM strains

The presence of the tagged mutants in the input and output pools was detected by PCR essentially as described [51]. All bacterial colonies obtained with each of the input and output pools were washed off from agar plates with 1 ml THY broth. A portion of the bacterial suspension (100 µl) was used to inoculate 10 ml THY broth to prepare bacterial genomic DNA by phenol-chloroform extraction [48]; the rest was stored at −80°C as a backup source. Each input and output DNA template was tested in 55 PCR reactions, each of which contained a tag-specific primer (not shown) and a common primer based on the plasmid backbone (Pr385). The tag-specific primers were synthesized based on our DNA sequence information for each of the tags [51]. To compare the amplification profiles of the input and output pools for the same mutant sets, PCR products were separated in 1.2% agarose gels and visualized by staining agarose gels with ethidium bromide (2 µg/ml). An STM strain was considered to be attenuated for infection when no or substantially decreased PCR products were observed from the output pools from both ears of at least two infected chinchillas (n = 4) or from nasal lavages of at least three mice compared with a positive PCR signal for the corresponding input pools.

Plasmid insertion sites in ST556 genome were determined by DNA sequencing of bacterial genomic DNA using a plasmid-based primer Pr289 as described [51]. The resulting DNA sequences were used to perform homology searches against the GenBank databases. DNA and protein sequence analyses were performed using the DNASTAR Lasergene v7.1 for Macintosh (Madison, WI).

### Construction of pneumococcal deletion mutants

Gene deletions were generated in strain ST556 by allelic exchange as described [48], [52]. Briefly, the up- and down-stream regions of target genes were PCR amplified from ST556 genomic DNA with primer pairs Pr1448/Pr1449 and Pr1450/Pr1451 for *tetM*, Pr1437/Pr1438 and Pr1439/Pr1440 for SP0308, Pr1515/Pr1516 and Pr1517/Pr1518 for SP1448, Pr1507/Pr1508 and Pr1509/Pr1510 for SP1732/*stkP*, Pr1537/Pr1538 and Pr1539/Pr1540 for *cysS*, Pr1541/Pr1542 and Pr1543/Pr1544 for *hisS*, Pr1553/Pr1554 and Pr1555/Pr1556 for *leuS,* Pr1545/Pr1546 and Pr1547/Pr1548 for *lysS*, and Pr1549/Pr1550 and Pr1551/Pr1552 for *thrS*. The PCR products were digested with *Xba*I (upstream regions) or *Xho*I (downstream regions), and ligated to the *Xba*I/*Xho*I-digested Janus cassette. The Janus cassette was amplified with primers Pr1097/Pr1098 from genomic DNA of pneumococcal strain ST588 [48]. To minimize sequence errors during PCR amplifications, a high fidelity Phusion DNA polymerase (New England Biolabs) was used for all PCR amplifications. The ligation mixtures were used to transform ST556 as described [48]. The transformants were selected for resistance to kanamycin and subsequently analyzed for the loss of target sequences in the ST556 chromosome by PCR amplification and DNA sequencing.

### Co-infections

Co-infection experiments were carried out essentially as described [51]. Broth cultures of wild type and isogenic mutants were separately incubated to OD_620_ ∼0.4, mixed in a 1∶1 ratio (v/v), and stocked with 15% glycerol (v/v). The CFU levels of the frozen stocks were enumerated in the presence (mutant only) or absence (mutant plus wild type) of kanamycin. The bacterial mixtures were diluted in PBS and used to infect the middle ears of chinchillas (∼4×10^4^ CFU/ear) and nasopharynx of mice (2×10^5^ CFU/mouse) as described above. The level of attenuation is expressed as the competitive index (CI), which is defined as the output CFU ratio (mutant/wild type) divided by the input CFU ratio (mutant/wild type).

### Statistical analysis

Statistical significance was determined by Student's *t* test. A *P* value of ≤0.05 was considered significant.

## Supporting Information

Figure S1 Deletion of aminoacyl-tRNA synthetase genes in strain ST556. (A). Schematic illustration of allelic exchange. The 5′ and 3′ flanking sequences of the aminoacyl-tRNA synthetase genes were PCR amplified, digested with Xba I and Xho I, respectively. The digested PCR products were ligated to the Xba I/Xho I-digested Janus cassette (carrying a kanamycin-resistance gene) to perform allelic exchange as described in the “Materials and Methods”. This procedure resulted in kanamycin-resistant strains ST1818 (cysS), ST1819 (hisS), ST1820 (lysS) (not shown), ST1821 (thrS), and ST1822 (leuS). (B). PCR amplification of the target gene loci. Each of the target aminoacyl-tRNA synthetase genes was amplified from the wild type and corresponding mutant strains with the flanking region-specific primers (e.g. Primers 1 and 4 in A). The PCR products were digested with Xho I to differentiate the mutant alleles from the wild type counterparts. Only the mutant alleles could be cut by Xho I. ST1595, a tetM mutant of ST556 (see Fig. 4), was used as a positive control. It should be noted that, in addition to the expected mutant allele, each of the cysS, hisS, thrS, and leuS mutants appeared to carry the wild type allele by an uncharacterized mechanism.
(6.48 MB TIF)

Table S1 (0.65 MB DOC)

Table S2 (0.30 MB DOC)

Table S3 (0.31 MB DOC)
