# Supplementary figures and images for: Genetic Requirement for Pneumococcal Ear Infection
Source: PLoS One. 2008 Aug 13;3(8):e2950. doi: 10.1371/journal.pone.0002950 (PMC2593789; doi:10.1371/journal.pone.0002950)

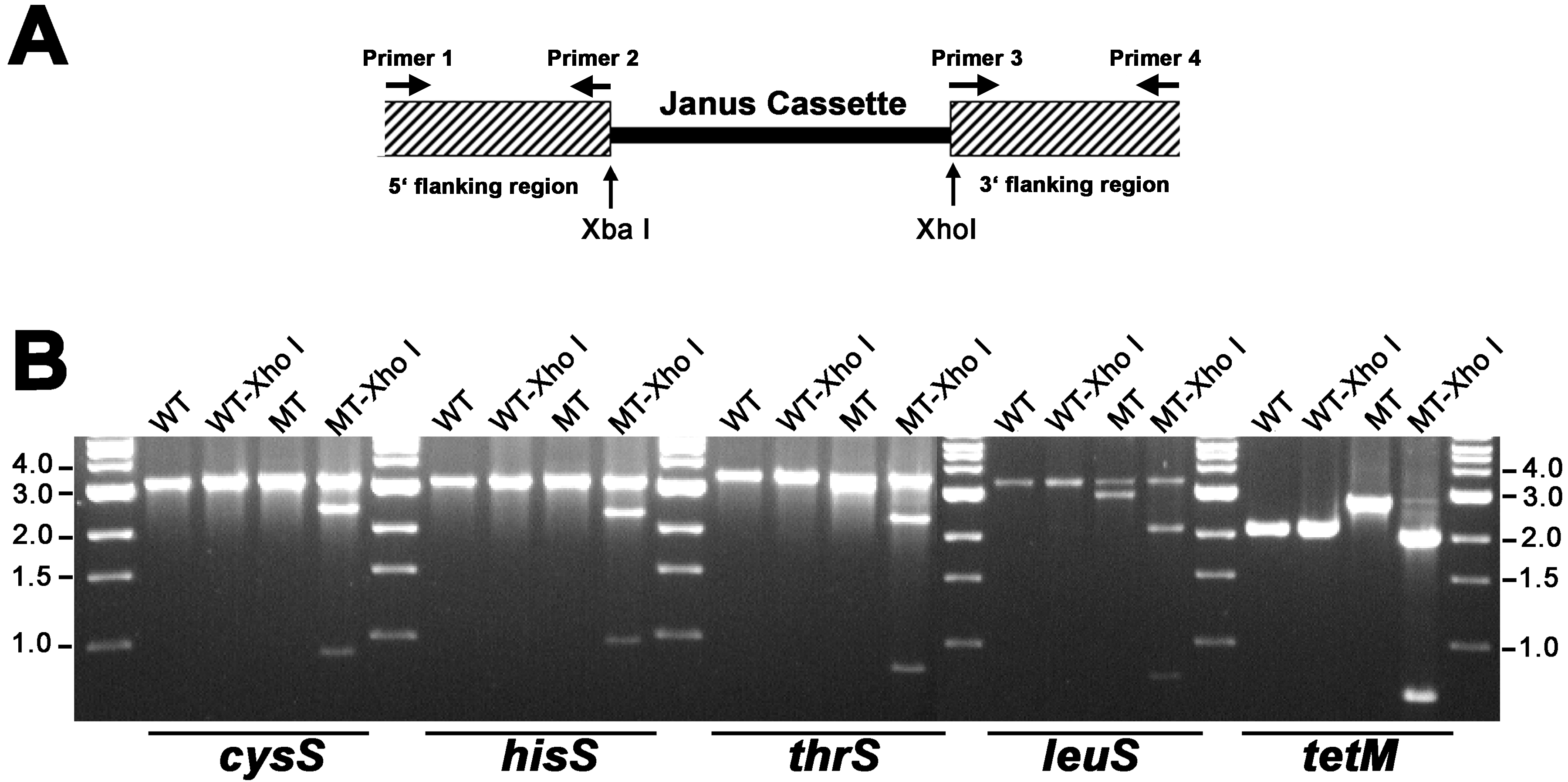

Supplement: Figure S1 — Deletion of aminoacyl-tRNA synthetase genes in strain ST556. (A). Schematic illustration of allelic exchange. The 5′ and 3′ flanking sequences of the aminoacyl-tRNA synthetase genes were PCR amplified, digested with Xba I and Xho I, respectively. The digested PCR products were ligated to the Xba I/Xho I-digested Janus cassette (carrying a kanamycin-resistance gene) to perform allelic exchange as described in the “Materials and Methods”. This procedure resulted in kanamycin-resistant strains ST1818 (cysS), ST1819 (hisS), ST1820 (lysS) (not shown), ST1821 (thrS), and ST1822 (leuS). (B). PCR amplification of the target gene loci. Each of the target aminoacyl-tRNA synthetase genes was amplified from the wild type and corresponding mutant strains with the flanking region-specific primers (e.g. Primers 1 and 4 in A). The PCR products were digested with Xho I to differentiate the mutant alleles from the wild type counterparts. Only the mutant alleles could be cut by Xho I. ST1595, a tetM mutant of ST556 (see Fig. 4), was used as a positive control. It should be noted that, in addition to the expected mutant allele, each of the cysS, hisS, thrS, and leuS mutants appeared to carry the wild type allele by an uncharacterized mechanism. (6.48 MB TIF) [file pone.0002950.s001.tif]
