# Supplementary material for: Genetic Requirement for Pneumococcal Ear Infection
Source: PLoS One. 2008 Aug 13;3(8):e2950. doi: 10.1371/journal.pone.0002950 (PMC2593789; doi:10.1371/journal.pone.0002950)
Supplement: Table S1 — (0.65 MB DOC) [file pone.0002950.s002.doc]

**Table S1 *– S. pneumoniae* ST556 genes essential for ear infection identified by STM**

| **Mutant ID^1^** | **TIGR4 ID^2^** | **Gene^3^** | **Description of disrupted gene^3^** | **NC STM^4^** | **Functional group^3^** | **Ref.^5^** |
| --- | --- | --- | --- | --- | --- | --- |
| 66A12 | SP0120 | *gidA* | Glucose-inhibited division protein A | **-** | Cell division |  |
| 56E09 | SP0571 |  | Cell filamentation protein | **-** | Cell division | (10) |
| 81E04 | SP0803 | *rodA* | Rod shape-determining protein | **+** | Cell division |  |
| 82E02 | SP1661 | *divIVA* | Cell division protein DivIVA | **-** | Cell division |  |
| 28D07 | NA |  | Zinc metalloprotease | **-** | Cellular processes |  |
| 61A12 | SP0081 |  | Glycosyl transferase | **-** | Cellular processes |  |
| 77B11 | SP0104 |  | Haloacid dehalogenase-like family hydrolase | **-** | Cellular processes |  |
| 60D01 | SP0106 | *sdhB* | Iron-sulfur-dependent L-serine dehydratase, β subunit | **-** | Cellular processes |  |
| 44C07 | SP0121 |  | Metallo-β-lactamase superfamily protein | **-** | Cellular processes | (10) |
| 07B09 | SP0289 |  | Dihydropteroate synthase | **-** | Cellular processes |  |
| 70C11 | SP0338 | *clpL* | ATP-dependent Clp protease, ATP-binding subunit | **-** | Cellular processes | (10) |
| 13A04 | SP0641 | *prtA* | Subtilase family serine protease | **-** | Cellular processes | (10) |
| 46E01 | SP0659 | *smsB* | Thioredoxin family enzyme | **-** | Cellular processes | (11) |
| 64E02 | SP0762 | *metK* | S-adenosylmethionine synthetase | **-** | Cellular processes |  |
| 38C08 | SP0829 | *deoB* | Phosphopentomutase | **-** | Cellular processes | (10) |
| 71E07 | SP0923 |  | Haloacid dehalogenase-like hydrolase | **-** | Cellular processes |  |
| 56A03 | SP0931 | *proB* | Glutamate 5-kinase | **+** | Cellular processes |  |
| 78F04 | SP1160 |  | Lipoate-protein ligase | **-** | Cellular processes |  |
| 95E01 | SP1201 |  | Serine/threonine protein phosphatase | **-** | Cellular processes |  |
| 28E04 | SP1238 | *uvrB* | Excinuclease ABC, subunit B | **-** | Cellular processes |  |
| 59D10 | SP1276 | *carA* | Carbamoyl-phosphate synthase, small subunit | **-** | Cellular processes |  |
| 92C07 | SP1450 |  | NnaC-like hydrolase | **+** | Cellular processes |  |
| 18E01 | SP1472 |  | Oxidoreductase | **-** | Cellular processes |  |
| 67A08 | SP2026 |  | Iron-containing alcohol dehydrogenase | **-** | Cellular processes |  |
| 62F08 | SP2157 |  | Iron-containing alcohol dehydrogenase | **-** | Cellular processes |  |
| 71B11 | NA | *glf* | UDP-galactopyranose mutase | **-** | Cellular processes |  |
| 74D03 | NA | *tetM* | Tetracycline resistance protein | **-** | Drug resistance |  |
| 72E04 | SP0251 | *smmF* | Formate acetyltransferase | **+** | Energy metabolism | (10, 11) |
| 48C09 | SP0303 | *bglA-1* | 6-phospho-β-glucosidase | **+** | Energy metabolism |  |
| 87E02 | SP1507 | *atpC* | ATP synthase F1, ε subunit | **+** | Energy metabolism |  |
| 28E11 | SP0054 | *purK* | Phosphoribosylaminoimidazole carboxylase, ATPase subunit | **+** | Nucleic acid processing |  |
| 22D07 | SP0180 |  | DNA-3-methyladenine glycosylase | **-** | Nucleic acid processing |  |
| 32D02 | SP0259 | *ruvB* | Holliday junction DNA helicase | **-** | Nucleic acid processing |  |
| 06C08 | SP0406 |  | DNA mismatch binding protein MutS2 | **+** | Nucleic acid processing |  |
| 62C06 | SP0458 | *dinP* | DNA-damage inducible protein P | **-** | Nucleic acid processing |  |
| 83E01 | SP0611 | *recJ* | Single-stranded-DNA-specific exonuclease | **-** | Nucleic acid processing | (12) |
| 45E12 | SP1152 | *rexA* | Exonuclease RexA | **+** | Nucleic acid processing |  |
| 32B03 | SP1179 | *nrdE* | Ribonucleoside-diphosphate reductase 2, α subunit | **-** | Nucleic acid processing |  |
| 02A12 | SP1219 | *gyrA* | DNA gyrase, A subunit | **-** | Nucleic acid processing |  |
| 15A02 | SP1248 | *rnc* | Ribonuclease III | **-** | Nucleic acid processing |  |
| 51C02 | SP1263 | *topA* | DNA topoisomerase I | **+** | Nucleic acid processing |  |
| 93C08 | SP1523 |  | Snf2 family helicase | **+** | Nucleic acid processing |  |
| 04C11 | SP1697 | *recG* | ATP-dependent DNA helicase | **+** | Nucleic acid processing |  |
| 09A03 | SP0117 | *pspA* | Pneumococcal surface protein A | **+** | Surface | (10) |
| 75B06 | SP2190 | *cbpA* | Choline binding protein A | **-** | Surface | (10) |
| 01B07 | SP0082 |  | Cell wall-anchored family protein | **+** | Surface |  |
| 29E04 | SP0468 | *sortD* | Sortase | **-** | Surface | (10) |
| 29E05 | SP0845 |  | Lipoprotein | **-** | Surface |  |
| 78C01 | SP1004 | *phtE* | Pneumococcal histidine triad protein E | **-** | Surface |  |
| 68A12 | SP1447 |  | Membrane protein | **+** | Surface |  |
| 06E12 | SP1013 | *asd* | Aspartate-semialdehyde dehydrogenase | **+** | Synthesis, amino acid |  |
| 89E12 | SP1306 | *gdhA* | NADP-specific glutamate dehydrogenase | **+** | Synthesis, amino acid |  |
| 83E02 | SP1361 | *hom* | Homoserine dehydrogenase | **-** | Synthesis, amino acid |  |
| 07C06 | SP1373 | *tyrA* | Prephenate dehydrogenase | **-** | Synthesis, amino acid |  |
| 90B03 | SP0427 | *accA* | Acetyl-CoA carboxylase, carboxyl transferase, α subunit | **-** | Synthesis, fatty acid |  |
| 51C05 | SP0046 | *purF* | Amidophosphoribosyltransferase | **-** | Synthesis, nucleic acid |  |
| 43B09 | SP0056 | *purB* | Adenylosuccinate lyase | **-** | Synthesis, nucleic acid | (11) |
| 63E01 | SP1445 | *guaA* | GMP synthase | **-** | Synthesis, nucleic acid |  |
| 35B11 | SP0100 |  | Transcriptional regulator | **-** | Transcription | (10) |
| 78E01 | SP0461 | *rlrA* | Transcriptional regulator | **-** | Transcription | (10) |
| 33B07 | SP0501 |  | Transcriptional regulator, MerR family | **-** | Transcription |  |
| 55A03 | SP0603 | *vncR* | DNA-binding response regulator | **-** | Transcription |  |
| 77E07 | SP0743 |  | Transcriptional regulator,TetR family | **-** | Transcription |  |
| 94E07 | SP1073 | *rpoD* | RNA polymerase sigma-70 factor | **-** | Transcription |  |
| 60D02 | SP1234 |  | Transcriptional regulator, biotin repressor family | **-** | Transcription |  |
| 81C05 | SP1362 | *mecA* | Transcriptional regulator | **+** | Transcription |  |
| 39B06 | SP1433 |  | Transcriptional regulator, AraC family | **-** | Transcription | (10) |
| 80F05 | SP1446 |  | Transcriptional regulator, GntR family | + | Transcription |  |
| 41B10 | SP1854 | *galR* | Galactose operon repressor | + | Transcription | (10) |
| 46E07 | SP2192 | *hk06* | Sensor histidine kinase | - | Transcription |  |
| 55B03 | SP0128 |  | Ribosomal-protein-alanine acetyltransferase | + | Translation | (10) |
| 25A03 | SP0254 | *leuS* | Leucyl-tRNA synthetase | - | Translation | (10) |
| 11B06 | SP0557 | *rbfA* | Ribosome-binding factor A | + | Translation |  |
| 46E12 | SP0591 | *cysS* | Cysteinyl-tRNA synthetase | - | Translation |  |
| 20C06 | SP0713 | *lysS* | Lysyl-tRNA synthetase | - | Translation |  |
| 05C08 | SP1020 | *prfA* | Peptide chain release factor 1 | - | Translation |  |
| 32B11 | SP1631 | *thrS* | Threonyl-tRNA synthetase | - | Translation |  |
| 28A01 | SP2078 | *argS* | Arginyl-tRNA synthetase | - | Translation |  |
| 53D06 | SP2121 | *hisS* | Histidyl-tRNA synthetase | - | Translation |  |
| 43F04 | SP0151 |  | Amino acid ABC transporter, ATP-binding protein | + | Transport |  |
| 51A08 | SP1116 |  | ABC transporter peamease protein | **-** | Transport |  |
| 36C06 | SP1166 |  | MATE efflux family protein | **+** | Transport |  |
| 61D02 | SP1357 |  | ABC transporter, ATP-binding protein | **+** | Transport |  |
| 83D09 | SP1434 |  | ABC transporter, ATP-binding protein | **-** | Transport | (10) |
| 95F04 | SP1957 |  | ABC transporter, ATP-binding protein | **+** | Transport |  |
| 75E09 | SP0042 | *comA* | Competence factor transporter, ATP-binding protein | **-** | Transport, amino acid |  |
| 60E05 | SP0453 |  | ABC transporter, amino acid-binding protein | **+** | Transport, amino acid |  |
| 79D02 | SP0708 |  | ABC transporter,amino acid-binding protein | **-** | Transport, amino acid |  |
| 78C03 | SP0709 |  | ABC transporter, ATP-binding protein | **-** | Transport, amino acid |  |
| 88A12 | SP0750 | *livH* | Branched-chain amino acid ABC transporter permease | **-** | Transport, amino acid |  |
| 88C08 | SP0824 |  | Amino acid ABC transporter, ATP-binding protein | **-** | Transport, amino acid |  |
| 81E07 | SP1891 | *amiA* | ABC transporter, oligopeptide-binding protein A | **+** | Transport, amino acid |  |
| 11D03 | SP2150 | *argF* | Ornithine carbamoyltransferase | **-** | Transport, amino acid |  |
| 14C08 | SP2152 |  | Arginine-ornithine antiporter | **+** | Transport, amino acid |  |
| 12E12 | SP2153 |  | Dipeptidase | **-** | Transport, amino acid |  |
| 19A03 | SP0483 |  | Cobalt ABC transporter, ATP-binding protein | **-** | Transport, ion | (11) |
| 63D08 | SP0729 | *ctpA* | Copper transporting ATPase, putative | **+** | Transport, ion | (10) |
| 64E12 | SP0241 | *pitA* | Iron ABC transporter, permease | **+** | Transport, iron |  |
| 69E07 | SP0723 | *thiW* | ThiW protein | **-** | Transport, nucleic acid |  |
| 54D08 | SP1286 | *uraA* | Uracil permease | **-** | Transport, nucleic acid | (10) |
| 17A03 | SP0057 | *strH* | β-N-acetylhexosaminidase | **+** | Transport/utilization, sugar | (10) |
| 14D03 | SP0308 |  | Cellobiose-specific PTS system IIA component | **-** | Transport/utilization, sugar |  |
| 76D09 | SP0310 |  | Cellobiose-specific PTS system, IIC component | **+** | Transport/utilization, sugar |  |
| 36B03 | SP0315 |  | Cellobiose-specific PTS system, IIC component | **-** | Transport/utilization, sugar |  |
| 23D02 | SP0318 |  | PfkB family carbohydrate kinase | **-** | Transport/utilization, sugar |  |
| 67A03 | SP0323 |  | PTS system, IIB component | **-** | Transport/utilization, sugar | (11) |
| 71D07 | SP0478 | *lacE-1* | Lactose-specific IIBC components | **+** | Transport/utilization, sugar | (10) |
| 32C08 | SP0498 | *endoD* | Endo-β-N-acetylglucosaminidase | **-** | Transport/utilization, sugar |  |
| 46D06 | SP0648 | *bgaA* | β-galactosidase | **-** | Transport/utilization, sugar | (10) |
| 51D02 | SP1190 | *lacD* | 1,6-diphosphate aldolase | **-** | Transport/utilization, sugar |  |
| 29C01 | SP1693 | *nanA* | Neuraminidase A | **-** | Transport/utilization, sugar |  |
| 18B03 | SP1796 |  | ABC transporter, sucrose-binding protein | **+** | Transport/utilization, sugar |  |
| 67B03 | SP2145 | *smuD* | α-1,2-mannosidase | **-** | Transport/utilization, sugar | (10, 11) |
| 04E02 | NA |  | Hypothetical protein | **-** | Unknown |  |
| 15B06 | NA |  | Hypothetical protein | **-** | Unknown |  |
| 20E07 | NA |  | No homologue | **-** | Unknown |  |
| 21B03 | NA |  | No homologue | **-** | Unknown |  |
| 28F04 | NA |  | No homologue | **-** | Unknown |  |
| 29A12 | NA |  | No homologue | **-** | Unknown |  |
| 38C11 | NA |  | No homologue | **-** | Unknown |  |
| 44A12 | NA |  | No homologue | **-** | Unknown |  |
| 54C11 | NA |  | No homologue | **-** | Unknown |  |
| 55E11 | NA |  | No homologue | **-** | Unknown |  |
| 56A12 | NA |  | No homologue | **-** | Unknown |  |
| 60F05 | NA |  | No homologue | **-** | Unknown |  |
| 69E02 | NA |  | No homologue | **-** | Unknown |  |
| 71D06 | NA |  | No homologue | **-** | Unknown |  |
| 74C11 | NA |  | No homologue | **-** | Unknown |  |
| 80D02 | NA |  | No homologue | **-** | Unknown |  |
| 82C5 | NA |  | No homologue | **-** | Unknown |  |
| 82E07 | NA |  | No homologue | **-** | Unknown |  |
| 84D01 | NA |  | No homologue | **-** | Unknown |  |
| 88E02 | NA |  | No homologue | **-** | Unknown |  |
| 95C04 | NA |  | No homologue | **-** | Unknown |  |
| 96B10 | NA |  | No homologue | **-** | Unknown |  |
| 06B07 | NA |  | Hypothetical protein | **+** | Unknown |  |
| 12D02 | NA |  | Hypothetical protein | **+** | Unknown |  |
| 22B10 | NA |  | No homologue | **+** | Unknown |  |
| 28B07 | NA |  | No homologue | **+** | Unknown |  |
| 32C06 | NA | *H10* | Otitis media-associated gene H10 | **+** | Unknown |  |
| 35C05 | NA |  | No homologue | **+** | Unknown |  |
| 56C05 | NA |  | No homologue | **+** | Unknown |  |
| 63A12 | NA |  | No homologue | **+** | Unknown |  |
| 69D07 | NA |  | No homologue | **+** | Unknown |  |
| 75C05 | NA |  | No homologue | **+** | Unknown |  |
| 89A03 | NA |  | No homologue | **+** | Unknown |  |
| 89B07 | NA |  | No homologue | **+** | Unknown |  |
| 95B06 | NA |  | No homologue | **+** | Unknown |  |
| 18D03 | SP0159 |  | Hypothetical protein | **-** | Unknown |  |
| 43C06 | SP0239 |  | Hypothetical protein | **-** | Unknown |  |
| 61B03 | SP0490 |  | Hypothetical protein | **+** | Unknown |  |
| 87A03 | SP0495 |  | Transposase IS1380-Spn1-like | **-** | Unknown |  |
| 81E05 | SP0592 |  | Leucine-rich protein | **-** | Unknown |  |
| 69B01 | SP0625 |  | Hypothetical protein | **-** | Unknown |  |
| 81D02 | SP0866 |  | Hypothetical protein | **-** | Unknown |  |
| 49A12 | SP0899 |  | Hypothetical protein | **-** | Unknown |  |
| 73E04 | SP0969 | *era* | GTP-binding protein Era | **-** | Unknown |  |
| 69F08 | SP1026 |  | Hypothetical protein | **+** | Unknown |  |
| 23B03 | SP1027 |  | Hypothetical protein | **-** | Unknown |  |
| 94C03 | SP1030 |  | Hypothetical protein | **-** | Unknown | (10) |
| 94B04 | SP1041 |  | Hypothetical protein | **-** | Unknown | (10) |
| 64D12 | SP1127 |  | Hypothetical protein | **+** | Unknown | (10) |
| 63C09 | SP1448 |  | Hypothetical protein | **-** | Unknown |  |
| 46D02 | SP1451 |  | Hydrolases | **-** | Unknown |  |
| 59A12 | SP1453 |  | CapA-like protein | **+** | Unknown |  |
| 39D02 | SP2057 |  | Hypothetical protein | **-** | Unknown |  |
| 55C05 | SP2140 |  | Hypothetical protein | **-** | Unknown |  |
| 45D09 | SP2155 |  | Hypothetical protein | **-** | Unknown |  |
| 89B07 | SP2191 |  | Hypothetical protein | **-** | Unknown |  |

^1^A single mutant for each ORF is shown when multiple independent mutants were identified for the ORF.

^2^The ORF designations are adopted from the annotations of the TIGR4 genome (accession AE005672). The ORFs unidentifiable in the TIGR4 genome are listed as NA (none applicable).

^3^The gene designations and functions are adopted from the annotated genome sequences of the TIGR4 genome (accession AE005672) and available literature.

^4^The attenuation status in the nasal colonization (NC) screen: +, attenuated; -, unattenuated.

^5^The ORFs identified in the previous three *S. pneumoniae* STM studies are referenced whenever possible. Direct comparison with the genes reported by Polissi et al. (12) and Lau et al. (11) was incomplete compared due to the lack of sufficient information.
