# Supplementary material for: Genetic Requirement for Pneumococcal Ear Infection
Source: PLoS One. 2008 Aug 13;3(8):e2950. doi: 10.1371/journal.pone.0002950 (PMC2593789; doi:10.1371/journal.pone.0002950)
Supplement: Table S2 — (0.30 MB DOC) [file pone.0002950.s003.doc]

**Table S2 *-* ST556 genes essential for nasal colonization but not for ear infection**

| **Mutant ID** | **TIGR4 ID** | **Gene** | **Description of disrupted gene** | **Functional group** | **Ref.** |
| --- | --- | --- | --- | --- | --- |
| 41E12 | SP0274 | *polC* | DNA polymerase III, alpha subunit | DNA processing | (10) |
| 73B04 | SP0149 |  | Lipoprotein | Surface |  |
| 42C09 | SP0436 | *gatB* | Glutamyl-tRNA(Gln) amidotransferase B subunit | Translation |  |
| 25A02 | SP0674 |  | Ribonuclease Z (tRNA processing) | Translation |  |
| 90E09 | SP1716 |  | ABC transporter, ATP-binding protein | Transport | (10) |
| 02A08 | SP1001 |  | ABC transporter, amino acid permease family | Transport, amino acid |  |
| 76D01 | SP0065 | *agaS* | Sugar isomerase domain protein | Transport/utilization, sugar |  |
| 94A12 | SP0322 |  | Glucuronyl hydrolase | Transport/utilization, sugar |  |
| 10D09 | NA |  | Hypothetical protein | Unknown | (10) |
| 74C03 | NA |  | No homologue | Unknown |  |
| 10B09 | SP0719 |  | Hypothetical protein | Unknown |  |
| 11A03 | SP2054 |  | Hypothetical protein | Unknown |  |

The result was arranged as in Table S1.
