# Supplementary material for: Genetic Requirement for Pneumococcal Ear Infection
Source: PLoS One. 2008 Aug 13;3(8):e2950. doi: 10.1371/journal.pone.0002950 (PMC2593789; doi:10.1371/journal.pone.0002950)
Supplement: Table S3 — (0.31 MB DOC) [file pone.0002950.s004.doc]

**Table S3**. **The primers used in this study**

| **Primer** | **Sequence** |
| --- | --- |
| Pr213 | 5'-TACCTACAACCTCAAGCT-3' |
| Pr214 | 5'-TACCCATTCTAACCAAGC-3' |
| Pr289 | 5’-AGACCATTCATGTTGTTGCTCAGG-3’ |
| Pr385 | 5'-CAGGAGTCCAAATACCAGAGAATGTT-3' |
| Pr1097 | 5’-GAGATCTAGAACCGTTTGATTTTTAATGGATAATG-3’ |
| Pr1098 | 5’-GAGACTCGAGCCTTTCCTTATGCTTTTGGAC-3’ |
| Pr1393 | 5'-GTTCGTTGATTTTCCATTAGTTGA-3' |
| Pr1394 | 5'-AATTGGGGAGGTTGGTATGTAAGC-3' |
| Pr1437 | 5'-ACGGAGTTTGGGAAGCGAGTA-3’ |
| Pr1438 | 5'-ACACTCTAGATTCTGCATCATCAAAACTACCTTC-3' |
| Pr1439 | 5'-ATCGCTCGAGGCAAGAATACGCAAGTGGAGT-3' |
| Pr1440 | 5'-GCACGTACTACTTTAAACTGAGCA-3' |
| Pr1448 | 5'-CGGGATCCCCTTTTGGGCTTTTGAATGGA-3' |
| Pr1449 | 5’-ACACTCTAGACCGTGATAAACAGGGAACAGG-3’ |
| Pr1450 | 5'-ATCGCTCGAGAGCTCGGTTTCTCTTGGATACT-3' |
| Pr1451 | 5'-CGGGATCCAATCATTTTCACACCCTTTCGTCT-3' |
| Pr1507 | 5'-AAAAAGGCAGTTGTTCGGTAGG-3' |
| Pr1508 | 5'-ACACTCTAGACTACAAACCTAAAACAACTTCAGC-3' |
| Pr1509 | 5'-CGGGATCCTCGAGATCATTCTGCATCCTCCTCGTT-3' |
| Pr1510 | 5'-CGGATCCAGGATATCATGAAAGACGGCTGTA-3' |
| Pr1515 | 5'-AAGGAAAGCTGTATAGGCAAAGAC-3' |
| Pr1516 | 5'-ACACTCTAGACCTATCGAATCGTACCTATGAAAA-3' |
| Pr1517 | 5'-ATCGCTCGAGCATGCTCTGGGTCACTCTGG-3' |
| Pr1518 | 5'-GGTTTTAGAAGAAGCTCCCAGTA-3' |
| Pr1537 | 5'-AGAAACAAGTTAAGCCAAGAGGAG-3' |
| Pr1538 | 5'-ACACTCTAGAATACATCTTGATCTTGCCGTCCTC-3' |
| Pr1539 | 5'-ATCGCTCGAGCAAGGATGGAGTGAGGTGGAC-3' |
| Pr1540 | 5'-AACCTGACCCAACATGATAGAAAG-3' |
| Pr1541 | 5'-ATGATTTGATGGAGGCTTATTCTT-3' |
| Pr1542 | 5'-ACACTCTAGATTTTGGTTTTTGTAATTTCATAGG-3' |
| Pr1543 | 5'-ATCGCTCGAGGACAATCAGCCAAAACTTCTCAG-3' |
| Pr1544 | 5'-TAGGTCCATCCCAATCAGTAGAAA-3' |
| Pr1545 | 5'-GAAGAACCACATAGCGAAAGACCT-3' |
| Pr1546 | 5'-ATCGCTCGAGACGATCTGCTGGTCATTTAGTTC-3' |
| Pr1547 | 5'-ACACTCTAGATGCTCCTCACTGATACAACAACTA-3' |
| Pr1548 | 5'-CAAGGAGATGAAGGAGGGATTT-3' |
| Pr1549 | 5'-CATCTTATTTTTCACTTGGGACAT-3' |
| Pr1550 | 5'-ACACTCTAGATCCTAGCTGATATCGCCAACA-3' |
| Pr1551 | 5'-ATCGCTCGAGCTGGGAAAAGACGACGAG-3' |
| Pr1552 | 5'-GCTGGAAACGCATAGGAA-3' |
| Pr1553 | 5'-TTCATAAATCAAAAAGCGACAT-3' |
| Pr1554 | 5'-ACACTCTAGATGGCACTTCAGCTTCATAGG-3' |
| Pr1555 | 5'-ATCGCTCGAGTGGCAAACAGTCGCAGAA-3' |
| Pr1556 | 5'-CTGGATTGAATACGAGGATAAGT-3' |
